# Supplementary material for: The interactome of intact mitochondria by cross-linking mass spectrometry provides evidence for coexisting respiratory supercomplexes
Source: Mol Cell Proteomics. 2017 Dec 8;17(2):216–32. doi: 10.1074/mcp.RA117.000470 (PMC5795388; doi:10.1074/mcp.RA117.000470)
Supplement: Supplemental Data [file supp_RA117.000470_133922_0_supp_23978_fzffwf.zip › spectra_annotation/mito_DR_spectra_annotation/390-1-7-1-8-1.pdf]

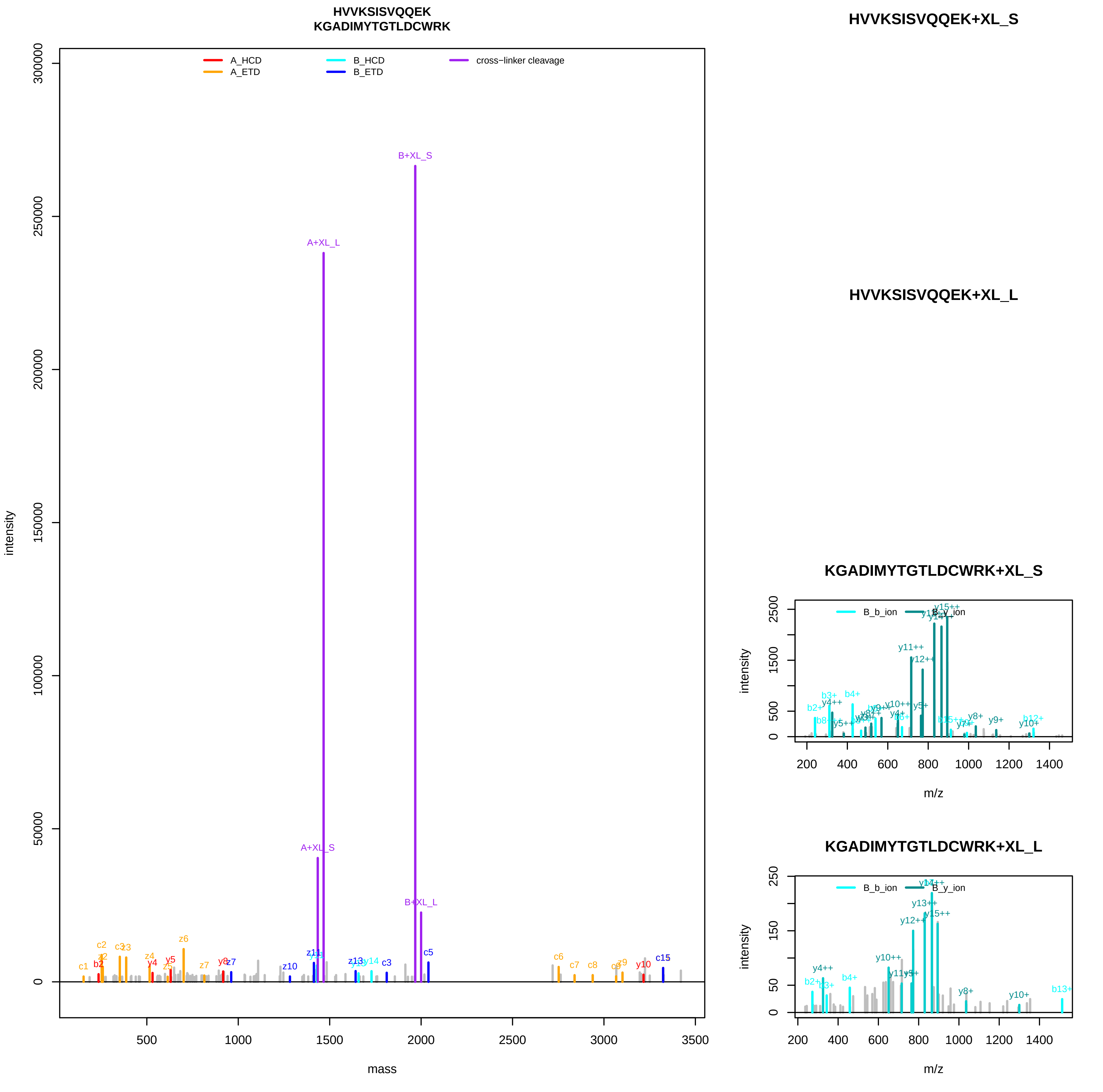
HVVKSISVQQEK
KGADIMYTGTLDCWRK
A_HCD
A_ETD
B_HCD
B_ETD
cross−linker cleavage
B+XL_S
A+XL_L
A+XL_S
B+XL_L
c1
b2
c2
c3
z4
y4
y5
z6
z7
y8
z7
z10
z11
z13
y14
c3
c5
c6
c7
c8
z9
y10
c15
intensity
mass
0
50000
100000
150000
200000
250000
300000
500
1000
1500
2000
2500
3000
3500
HVVKSISVQQEK+XL_S
HVVKSISVQQEK+XL_L
KGADIMYTGTLDCWRK+XL_S
B_b_ion
B_y_ion
y15++
y11++
y12++
b3+
b4+
b2+
y4++
y10++
y5+
y8+
y9+
y10+
b12+
intensity
m/z
0
500
1500
2500
200
400
600
800
1000
1200
1400
KGADIMYTGTLDCWRK+XL_L
B_b_ion
B_y_ion
y14++
y13++
y15++
y12++
y10++
y4++
b2+
b3+
b4+
y8+
y10+
b13+
intensity
m/z
0
50
150
250
200
400
600
800
1000
1200
1400
